# The Role of Cannabinoid Type 2 Receptors in Parkinson’s Disease

**DOI:** 10.3390/biomedicines10112986

**Published:** 2022-11-20

**Authors:** Maria Sofia Basile, Emanuela Mazzon

**Affiliations:** IRCCS Centro Neurolesi “Bonino-Pulejo”, Via Provinciale Palermo, Contrada Casazza, 98124 Messina, Italy

**Keywords:** cannabinoid type 2 receptors, Parkinson’s disease, biomarkers, therapeutic targets

## Abstract

Parkinson’s disease (PD) is the second most frequent neurodegenerative disease and currently represents a clear unmet medical need. Therefore, novel preventive and therapeutic strategies are needed. Cannabinoid type 2 (CB2) receptors, one of the components of the endocannabinoid system, can regulate neuroinflammation in PD. Here, we review the current preclinical and clinical studies investigating the CB2 receptors in PD with the aim to clarify if these receptors could have a role in PD. Preclinical data show that CB2 receptors could have a neuroprotective action in PD and that the therapeutic targeting of CB2 receptors could be promising. Indeed, it has been shown that different CB2 receptor-selective agonists exert protective effects in different PD models. Moreover, the alterations in the expression of CB2 receptors observed in brain tissues from PD animal models and PD patients suggest the potential value of CB2 receptors as possible novel biomarkers for PD. However, to date, there is no direct evidence of the role of CB2 receptors in PD. Further studies are strongly needed in order to fully clarify the role of CB2 receptors in PD and thus pave the way to novel possible diagnostic and therapeutic opportunities for PD.

## 1. Parkinson’s Disease (PD)

PD is the second most frequent neurodegenerative disease following Alzheimer’s disease, with a global prevalence of more than 6 million individuals that is expected to double over the next generation [1,2]. It has been shown that PD affects 1–2 per 1000 of the population at any time and 1% of the population above 60 years, thus indicating that PD prevalence increases with age [3]. The disease duration can last decades, with a typical presentation of a slow progression with accumulating disability for patients [4]. Therefore, to date, PD represents a mounting socioeconomic burden for society [4].

While less than 10% of PD cases show a strict familial etiology, the majority of them are sporadic and seem to be caused by susceptibility genes along with other factors, which could include age, gender, and environmental factors [5].

Age is the most important risk factor for PD, whereas the male gender is associated with a moderate risk [6]. Exposure to pesticides, high intake of dairy products, history of melanoma, traumatic brain injury, and depression have been associated with a higher risk of PD [7,8]. In particular, pesticides inhibit mitochondrial complex I, thus leading to mitochondrial dysfunction and death of dopaminergic neurons in the substantia nigra [9]. Instead, smoking, caffeine consumption, increased serum urate concentrations, physical activity, and use of non-steroidal anti-inflammatory drugs have been associated with decreased risk of PD [7,10].

The pathological hallmarks of PD are the loss of dopaminergic neurons in the substantia nigra pars compacta and the presence of Lewy bodies, which are intra-cytoplasmic inclusions consisting mainly of α-synuclein aggregations [6]. Most of the motor deficits present in PD patients are due to the progressive death of dopaminergic neurons in the substantia nigra pars compacta and to the subsequent loss of projections to the striatum [11]. Indeed, this neurodegeneration leads to dopamine depletion, facilitating the impairment of critical pathways linked with the regulation of voluntary movements, involving the basal ganglia, brainstem, cerebral cortex, and thalamus [11].

The most important molecular pathogenic mechanisms involved in PD are mitochondrial dysfunction, α-synuclein misfolding and aggregation, neuroinflammation, oxidative stress, and impairment of protein clearance, which is linked with deficient ubiquitin-proteasome and autophagy-lysosomal systems [12]. In addition, another mechanism involved in the pathogenesis of PD is excitotoxicity, that is, the pathological process through which neurons are damaged and killed following exaggerated stimulation of glutamatergic receptors by glutamate or similar substrates [13]. Moreover, neurogenesis, an important mechanism of brain development and plasticity, is impaired in PD [14].

In daily practice, the diagnosis of PD is clinical and relies on history taking and neurological examination [4]. According to the current criteria, PD is defined as the presence of bradykinesia along with either rest tremor, rigidity, or both [4]. In addition, the response of motor symptoms to levodopa is an important diagnostic characteristic of PD [6].

Even though PD is defined as a movement disorder, the clinical presentation is diversified and involves also different non-motor symptoms, such as depression, pain, memory loss, sleep disturbances, hyposmia, constipation, orthostatic hypotension, and urinary dysfunction [1,4]. 

To date, no efficacious neuroprotective or disease-modifying therapies have been found for PD [12]. The treatment of PD is symptomatic and levodopa represents the gold standard treatment of PD and the most efficacious drug for motor symptoms [6]. Nonetheless, in the long-term, treatment with levodopa becomes suboptimal owing to the appearance of motor fluctuations, including wearing-off and on-off phenomena, and dyskinesias (levodopa-induced dyskinesias or LIDs) [15]. Levodopa is the precursor to dopamine and can pass through the blood–brain barrier (BBB) and can be converted to dopamine in the residual dopaminergic neurons of the substantia nigra pars compacta [6,16]. In order to prevent its peripheral metabolism and to decrease the risk of nausea, levodopa is usually administered with aromatic acid decarboxylase inhibitors (carbidopa or benserazide) [12]. In addition to levodopa, there are different other drugs for the treatment of the motor symptoms related to PD, such as anticholinergics, amantadine, monoamine oxidase inhibitors, catechol-O-methyl transferase inhibitors, dopamine agonists, and istradefylline [12]. 

Anticholinergics are mainly used to decrease tremors, whereas amantadine, which is an N-methyl-D-aspartate-type (NMDA) glutamate receptor antagonist, is the most important drug for levodopa-related dyskinesia treatment [6,12].

Among the monoamine oxidase inhibitors, selegiline and rasagiline are most commonly used in early and mild PD, but they are also efficacious in patients with moderately advanced PD who show levodopa-related motor complications [12]. Instead, safinamide, another monoamine oxidase inhibitor, can rise the mean on time without troublesome dyskinesia and can decrease daily and morning off times [12]. 

Dopamine agonists, if administered early, can postpone levodopa-related complications (e.g., dyskinesias and motor fluctuation) [12].

Catechol-O-methyl transferase inhibitors can block the degradation of peripheral levodopa with an aim to extend the effectiveness of levodopa [12]. Thus, they can be used as adjunctive treatment for patients with levodopa-related motor fluctuations [12].

Instead, istradefylline, which is an adenosine A2 receptor antagonist, can be used as an adjunctive drug for levodopa/carbidopa in PD patients who show off episodes and can exert a moderate benefit in PD patients with levodopa-related motor fluctuations [12].

Nondopaminergic approaches, including selective serotonin reuptake inhibitors and cholinesterase inhibitors, are used for non-motor symptoms such as psychiatric symptoms and cognition [17]. In addition to pharmacologic approaches, there are also nonpharmacologic approaches, including exercise and physical, occupational, and speech therapies [17].

A possible approach to treat advanced PD is represented by deep brain stimulation, which consists of the use of chronic, high-frequency direct electrical current on a target that could be the subthalamic nucleus, the thalamus, or the globus pallidus internus [6].

To date, deep brain stimulation has mostly substituted ablative surgical approaches (e.g., thalamotomy, pallidotomy) [12]. 

Currently, different novel therapeutic strategies for PD are being investigated in clinical trials, including anti-α-synuclein aggregation therapy, convalescent plasma therapy, cell-based therapy, gene therapy, dopamine receptor agonists, serotonin receptor partial agonists or antagonists, monoamine reuptake inhibitors, muscarinic and nicotinic acetylcholine receptor agonists, NMDA receptor modulators, kinase inhibitors, myeloperoxidase inhibitors, anti-apoptotic drugs, adenosine A_2A_ receptor antagonists, antioxidants and botanical-based therapy, and others [18]. In particular, anti-α-synuclein aggregation therapies include monoclonal antibodies, vaccines, or small molecules, and the use of α-synuclein monoclonal antibodies to reduce the accumulation and spread of aggregated, toxic α-synuclein represents one of the most interesting strategies for potential neuroprotective or disease-modifying therapies for PD [12]. Moreover, plasma therapy is another potential therapy for PD since it is known that the infusion of the young plasma decrease α-synuclein and Lewy bodies [18]. Instead, cell therapy consists of the introduction of dopamine-producing cells into the brain via transplantation [18]. The development of cell-replacement therapies that include dopamine neurons derived from human pluripotent stem cells has shown different benefits in comparison with fetal cell-derived therapies [19]. Furthermore, gene therapy for PD treatment consists of genetically engineered therapeutic genes which actively replace, knockout, or correct defective genes in patients with PD [18].

Another possible interesting approach that has been suggested is the use of cannabinoids as a promising therapeutic strategy for PD treatment, thanks to their neuroprotective and motor symptom-modulating properties [20,21]. To date, medical cannabis has been legally approved in different countries for the treatment of PD patients [22,23]. In spite of the insufficiency of solid scientific evidence, patients with PD that use cannabis refer to positive effects on mood, memory, fatigue, obesity, sleep, pain, tremor, rigidity, and bradykinesia following its consumption [24]. 

However, overall, nowadays PD still represents a clear unmet medical need, not only owing to the absence of fully efficacious preventive and curative treatments, but also to the lack of reliable biomarkers for early diagnosis and management of the disease course [25,26]. Indeed, neurodegeneration in PD begins years prior to the clinical diagnosis and one of the biggest challenges of PD comprises the impotence of making a definitive diagnosis at early stages along with the difficulty in disease progression prediction, thus suggesting the need to identify early biomarkers for PD [26,27].

Therefore, further studies with the aim of discovering novel approaches to ameliorate the therapeutic outcome, alone or along with existing treatments, and to identify novel valid biomarkers are strongly needed [25,26]. 

## 2. The Cannabinoid Type 2 (CB2) Receptors in PD

Different preclinical and clinical studies have investigated the CB2 receptors in PD, suggesting their potential as possible biomarkers for PD and as promising therapeutic targets for alleviating parkinsonian symptoms and slowing disease development in PD patients [28,29]. Indeed, the activation of the CB2 receptors with selective agonists could have neuroprotective effects in the neurodegenerative processes of PD [30]. The CB2 receptor is one of the components of the endocannabinoid system, a system consisting of endogenous signals, receptors, and metabolic enzymes [31]. In particular, the endocannabinoid system is constituted by the two endocannabinoids 2-arachidonoylglycerol and anandamide, the cannabinoid type 1 (CB1) and CB2 receptors, and the endocannabinoid anabolic and catabolic enzymes [31]. Interestingly, the endocannabinoid signaling is implicated in the regulation of the homeostasis of the cell, tissue, organ, and organism as well as in synaptic plasticity, neurotransmitter release, cytokine release from microglia, and brain development, thus suggesting that it could be involved in different neurological diseases [31]. Considering that endocannabinoids activate various receptors and that their biosynthetic and catabolic pathways can be frequently in common with other mediators, it has been suggested that the system can be included in an expanded signaling system, known as the endocannabinoidome [31]. Several other receptors are involved in the endocannabinoidome, including transient receptor potential cation channel subfamily V member 1 (TRPV1), peroxisome proliferator-activated receptor-α (PPARα), peroxisome proliferator-activated receptor-γ (PPARγ) and the two orphan G protein-coupled receptors (GPCRs), GPR55, and GPR18 [31].

The CB2 receptor is encoded by the *CNR2* gene, which is located on 1p36.11 [32]. Genetic studies have revealed that CB2 receptors knockout (KO) mice showed enhanced microglial activation, neural pathology, and inflammation [2]. Two different human CB2 receptor isoforms (hCB2A and hCB2B) have been identified [33,34]. hCB2B was mostly observed in the spleen and leukocytes, whereas hCB2A expression was mainly detected in the testis and brain [33,34].

CB2 receptors, as well as CB1 receptors, are GPCRs, and can regulate different intracellular signal transduction pathways, including the inhibition of the production of cAMP, the activation of pERK and G protein-coupled Inward Rectifying K^+^-channels (GIRKs), and the recruitment of β-arrestin to the receptor [29,35]. In addition, CB2 receptors can modulate the ventral tegmental area dopamine neuron excitability via synaptic and intrinsic mechanisms, which include a decrease in presynaptic glutamate release and enhancement of postsynaptic neuronal M-currents [36]. 

Differently from CB1 receptors, which are the most abundant GPCRs in the brain, higher levels of CB2 receptors are mainly found in peripheral organs with immune function [29]. Although CB2 receptors were initially considered peripheral receptors, since earlier studies suggested that they were not present in the brain, recent studies have shown their presence in the central nervous system [29]. In particular, it has been demonstrated that CB2 receptors are expressed in microglia, astrocytes, and neurons in different areas, including the hippocampus, striatum, and brain stem [29,37].

It is worth mentioning that CB2 receptors are highly inducible [38]. In physiological conditions, the brain CB2 receptors are expressed at low levels, whereas in pathological conditions, including neurodegenerative diseases, their expression is enhanced and up-regulated [39]. 

Furthermore, it has been suggested that CB2 receptors could represent a promising therapeutic target not only in PD but also in different other neurodegenerative disorders, such as Alzheimer’s disease, Huntington’s disease, amyotrophic lateral sclerosis, and multiple sclerosis [2,30,34,40,41,42,43,44].

In particular, as regards PD, the activation of CB2 receptors might affect disease progression through the regulation of neurotransmission and neuronal function [21]. 

It might be hypothesized that CB2 receptors could alleviate excitotoxicity and attenuate oxidative damage in PD and could promote neural regeneration, thus delaying PD progression [21]. Interestingly, the most largely studied mechanism of neuroprotection involves the anti-inflammatory effects of the CB2 receptors [30].

Noteworthily, it has been shown that CB2 receptors are largely distributed in activated astrocytes and microglia and that, when activated, CB2 receptors regulate immune responses and inflammatory pathways; thus they have an important role in the regulation of neuroinflammation [21,45].

This is of particular relevance considering that neuroinflammation is a crucial component of PD pathogenesis [30].

**Figure 1:**
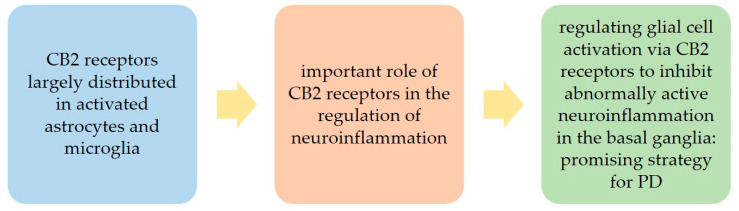
Potential value of the role of the cannabinoid type 2 (CB2) receptors in the regulation of neuroinflammation in Parkinson’s disease (PD).

The expression of CB2 receptors by glia allows them to contribute to the control by glial cells of neuronal homeostasis, survival, and integrity, in particular when glial cells become reactive [41]. Of note, it has been suggested that regulating glial cell activation via CB2 receptors to inhibit abnormally active neuroinflammation in the basal ganglia could be a promising strategy for PD (Figure 1) [21]. 

As regards the activated astrocytes, the beneficial effects resulting from CB2 receptor activation could be associated with the increase of their trophic role as well as the enhancement of the production of neurotrophins, anti-inflammatory mediators, and/or pro-survival factors, and the inhibition of the production of chemokines involved in neuronal damage [41]. These effects could depend on the activation of CB2 receptors, alone or along with CB1 receptors [41].

The beneficial effects of targeting CB2 receptors in activated microglia could be associated with the regulation of the production of TNF-α as well as of other microglia-derived neurotoxic factors and the regulation of migration and proliferation at lesion sites and of the balance of M1 vs. M2 phenotypes [41].

Moreover, it has been shown that cannabinoids could exert anti-inflammatory effects via CB2 receptors [21].

In addition to the interesting data above described that suggest a potentially important role of CB2 receptors in PD, another reason that led us to focus on this topic is that owing to the distribution of the CB2 receptors, drugs binding to CB2 receptors could exert a neuroprotective action without the central side effects characteristic of CB1 receptor ligands [34,46]. In particular, it has been found that CB1 receptor activation could be associated with severe psychiatric side effects, such as anxiety, depression, and suicidal thoughts [47]. Hence, selective CB2 receptor agonists could represent an alternative promising strategy in order to avoid the psychotropic effects linked with CB1 receptors [46,48].

Nonetheless, it should be noted that since the CB2 receptor is largely expressed in peripheral organs and in particular in the immune system, it might have some side effects, including immunosuppression [48].

Among the selective CB2 receptor agonists there are: AM1241, GW833972A, GW842166X, HU-308, JHW-133, NESS400, GSK554418A, phenyl morpholinyl analog, A-836339, and pyridine-based compounds [48]. In addition, β-caryophyllene (BCP) has CB2 receptor agonistic activity [48]. Moreover, among the other CB2 receptor ligand classes under investigation are tricyclic and bicyclic classical and non-classical cannabinoids derived from tetrahydrocannabinol (THC), 3-carbamoyl 2-pyridones, benzimidazoles, indole-based ligands, γ-carbolines, imidazoles, purine derivatives, pyridines, sulfamoylbenzamides, 1,4-diazepane carboxamides, thiophene amide derivatives, 1,8-naphthyridinone and dihydroquinoline-3-carboxamides [48]. 

Considering all these data, in this review, we describe the preclinical and clinical studies investigating CB2 receptors in PD, with the aim to clarify if these receptors could have a role in PD.

## 3. Preclinical Studies

### 3.1. Studies in PD Cellular Models

Different studies have investigated the CB2 receptors in PD cellular models. 

He et al. have demonstrated in vitro, in a PD cellular model obtained by the administration of 6-hydroxydopamine (6-OHDA) to PC12 cells, that the CB2 receptor-selective agonist AM1241 or the overexpression of Xist protected neuronal cells from death caused by 6-OHDA, and augmented Pitx3 expression [49].

Aymerich et al. have shown that the monoacylglycerol lipase (MAGL) inhibitor JZL184 exerted neuroprotective effects in an in vitro cellular PD model that consists of SH-SY5Y cells treated with 1-methyl-4-phenylpyridinium iodide (MPP+) [50]. Interestingly, the effect of JZL184 in cell survival was inhibited by the CB2 receptor inverse agonist AM630, whereas it was mimicked by the CB2 receptor agonist JWH133 [50]. Instead, the CB1 receptor antagonist Rimonabant did not influence cell survival induced by JZL184 [50]. Therefore, these data indicate that CB2 receptors can be involved in the neuroprotective effect of JZL184 [50]. 

Gugliandolo et al. have investigated the protective effects of cannabidiol (CBD), in an in vitro PD model obtained treating the retinoic acid (RA)-differentiated neuroblastoma SH-SY5Y cells with the toxin MPP+ [51]. CBD is an inverse agonist of CB2 receptors, a negative allosteric modulator of the CB1 receptors, and an agonist of TRPV1 receptors [51]. In addition, CBD can activate also the PPARγ, GPR3, GPR6, GPR12, GPR18, and GPR55 receptors, leading to different biochemical, molecular, and behavioral effects owing to the wide variety of receptors that it activates in the central nervous system [52]. In order to analyze the mechanisms of action of CBD and to discover the receptors implicated in the actions of CBD, TRPV1, CB1 and CB2 receptor antagonists were utilized in this study [51]. Interestingly, it has been shown that the protective action of CBD could involve the activation of ERK and AKT/mTOR pathways and that the ERK activation induced by CBD could be mediated by the interaction of TRPV1 and CB2 receptors with CBD [51]. In addition, it has been demonstrated that CBD decreased the MPP+-induced rise of the autophagic protein LC3 by TRPV1 and CB2 receptors [51]. Overall, treatment with CBD could be a potential preventive strategy for PD, since it can increase cell viability via the inhibition of apoptosis, the activation of the ERK and AKT/mTOR pathways, and the regulation of autophagy [51].

Burgaz and colleagues have explored the effects of VCE-004.8, the 3-hydroxyquinone derivative of CBD, which has agonist activity at the CB2 receptor besides its activity at the PPAR-γ receptor, in PD models obtained with the neurotoxin 6-OHDA [53]. They found that VCE-004.8 exerted neuroprotective effects in 6-OHDA-lesioned mice and, thus, they have investigated the mechanisms involved in these beneficial effects of VCE-004.8 by analyzing cell survival in SH-SY5Y cells exposed to 6-OHDA and by exploring the contribution of CB2 and PPAR-γ receptors using selective blockade with SR144528 and T0070907, respectively [53]. They have shown that VCE-004.8, at a concentration of 10 μM, exerted an important cytoprotective effect, which was completely reversed by the treatment with the antagonist of PPAR-γ receptors T0070907 and the CB2 receptor inverse agonist SR144528 [53,54]. The complete elimination of the cytoprotective effect only with the combination of T0070907 and SR144528 together indicate that both PPAR-γ and CB2 receptors contribute to the effect of VCE-004.8 [53]. However, while T0070907 alone only induced a partial reversal, SR144528 alone did not exert any effects, thus suggesting that PPAR-γ receptors are more relevant in comparison to CB2 receptors in the cytoprotective effect of VCE-004.8 [53]. 

Instead, Mnich et al. have explored the effects of anandamide, an endogenous cannabinoid that can bind and activate CB1, CB2, and TRPV1 receptors, on 6-OHDA-induced toxicity in rat adrenal phaeochromocytoma PC12 cells [55]. They have shown that anandamide was able to inhibit 6-OHDA-induced apoptosis and that the protection was not influenced by CB1, CB2, and TRPV1 receptor antagonists [55]. Therefore, according to these data, CB1, CB2, and TRPV1 receptors are not involved in the protective action of anandamide against 6-OHDA toxicity [55]. 

Overall, the in vitro studies in PD cellular models have shown the protective effects exerted by the CB2 receptor-selective agonist AM1241 and the involvement of CB2 receptors in the neuroprotective effects of JZL184, CBD, and VCE-004.8 [49,50,51,53]. Instead, it has been shown that CB2 receptors are not involved in the protective action of anandamide [55].

### 3.2. Studies in PD Animal Models

Different studies have explored the CB2 receptors in animal models of PD. 

Concannon et al. have shown that the striatal injection of 6-OHDA or lipopolysaccharide (LPS) in the neurotoxic 6-OHDA and inflammation-driven LPS PD rat models led to a marked striatal neuroinflammation, correlated with an upregulation in the expression of the CB2 receptor gene in the striatum [56]. In addition, this increase in the CB2 gene expression was significantly correlated with an increase in microglial activation [56]. Of note, the dysregulation in the endocannabinoid system was higher in the inflammation-driven LPS model than in the neurotoxic 6-OHDA model, showing a stronger increase in the CB2 gene expression and also an increase in the levels of the endocannabinoids anandamide and 2-arachidonylglycerol [56].

Moreover, Concannon et al. have also explored the changes in the endocannabinoid system in other two PD rat models, the environmental toxin rotenone model and the viral inflammation-driven polyinosinic:polycytidylic acid (Poly (I:C)) model [57]. They found that the administration of both rotenone and Poly (I:C) in the striatum caused a significant neuroinflammatory response, correlated with higher expression of the CB2 receptor in the striatum [57]. In particular, following the striatal administration of rotenone or Poly (I:C), CB2 receptor expression was found to be significantly upregulated both in the rotenone and Poly (I:C) models, whereas this rise was significantly associated with a rise in microglial activation in the rotenone model [57]. Of note, Poly (I:C) led to a higher increase in the expression of CB2 receptors, along with a downregulation in CB1 receptor expression and an increment in 2-arachidonylglycerol [57]. 

Overall, these studies have shown that the endocannabinoid system is differently dysregulated in different PD animal models and that upregulation of CB2 receptors, along with microglial activation, can occur in response to a Parkinsonian trigger with a neurotoxic, environmental, viral, or bacterial mechanism of action [56,57]. Furthermore, these studies have also shown a more important role of microglial CB2 receptor expression in inflammation-driven neurodegeneration compared with direct neurotoxin-driven neurodegeneration, thus suggesting that the CB2 receptor could be a promising target for anti-inflammatory disease modification in PD [56,57]. 

Different other studies have explored the regulation of CB2 receptors, as well as the effects of different CB2 receptor agonists and the effects of the genetic ablation of CB2 receptors.

Price and colleagues have investigated the effects of a non-selective cannabinoid receptor agonist, WIN55,212-2, in a PD animal model [58]. They have shown that the chronic administration of WIN55,212-2, at a dose of 4 mg/kg, intraperitoneal (i.p.) protected mouse nigrostriatal neurons from the neurodegenerative effects caused by 1-methyl-4-phenyl-1,2,3,6-tetrahydropyridine (MPTP) [58]. They have found that the neuroprotective action of WIN55,212-2 was independent of CB1 receptor activation [58]. Moreover, in agreement with the above-described studies, they have shown that three days after MPTP administration there were a significant microglial activation and an up-regulation of CB2 receptors in the ventral midbrain of the MPTP-treated mice [58]. Interestingly, treatment with WIN55,212-2 (4 mg/kg, i.p.) or with the CB2 receptor agonist JWH015 (4 mg/kg, i.p.), decreased the microglial activation induced by MPTP and this effect can be mediated by CB2 receptors [58]. On the other hand, genetic ablation of CB2 receptors exacerbated the MPTP systemic toxicity [58]. Therefore, these data suggest that agonism at CB2 receptors can protect against nigrostriatal degeneration induced by MPTP through the inhibition of microglial activation/infiltration, thus indicating the potential of CB2 receptors as a novel therapeutic target for PD [58]. 

In addition, Garcia et al. have studied the effects of Δ^9^-tetrahydrocannabivarin (Δ^9^-THCV), a phytocannabinoid able to block CB1 receptors and activate CB2 receptors, in rat and mouse PD models [59]. They found that the acute injection of Δ^9^-THCV (2 mg·kg^−1^) reduced the motor inhibition induced by 6-OHDA, possibly via alterations in glutamatergic transmission, whereas the chronic treatment of Δ^9^-THCV (2 mg·kg^−1^) decreased the loss of tyrosine hydroxylase-positive neurons induced by 6-OHDA in the substantia nigra thanks to its antioxidant activity [59]. Moreover, they have shown that the response of CB2 receptor-deficient mice to 6-OHDA was similar to that of wild-type (WT) animals [59]. Furthermore, it has been demonstrated that CB2 receptors were scarcely up-regulated in the substantia nigra of rats after 6-OHDA [59]. On the other hand, the substantia nigra of mice treated with LPS showed a higher up-regulation of CB2 receptors [59]. Moreover, Δ^9^-THCV preserved tyrosine hydroxylase-positive neurons in these animals and it is possible that this effect could be mediated by CB2 receptors since it was also induced by HU-308 (5 mg·kg^−1^), a selective CB2 receptor agonist, and CB2 receptor-deficient mice were more susceptible to LPS lesions [59].

Instead, Simkins et al. have evaluated whether CB1/CB2 KO mice could be more susceptible to MPTP owing to the absence of the protective effects of signaling via CB1 and CB2 receptors [60]. They have shown that CB1/CB2 KO mice were equally susceptible to MPTP, thus indicating that the absence of CB1 and/or CB2 signaling is neither protective nor harmful for the susceptibility of nigrostriatal dopamine neurons to MPTP toxicity [60]. 

In addition, Gómez-Gálvez et al. have studied the CB2 receptors in an inflammatory model of PD obtained via intrastriatal injections of LPS in mice [61]. In particular, in accordance with the previous studies, they have observed elevated levels of the CB2 receptor, using qRT-PCR, in the striatum and substantia nigra of LPS-lesioned mice and also a rise in the immunostaining for this receptor in the LPS-lesioned striatum [61]. In addition, they have shown a significant rise in CD68 immunostaining, used to identify the activation of microglia and the infiltration of peripheral macrophages, in these brain structures after the LPS insult, which was higher in CB2 receptor-deficient mice as regards the substantia nigra [61]. Moreover, the authors have shown that the pharmacological activation of CB2 receptors with the selective CB2 receptor agonist HU-308 (5 mg/kg, i.p.) was able to reverse the LPS-induced elevation of CD68 immunofluorescence in the striatum as well as the parallel decrease in tyrosine hydroxylase immunofluorescence in the substantia nigra [61]. Furthermore, it has been shown that LPS increased the gene expression of various pro-inflammatory mediators in the striatum and the substantia nigra [61]. Instead, the selective activation of CB2 receptors decreased some of these mediators, such as inducible nitric oxide synthase (iNOS), despite only in the striatum [61]. 

Moreover, Palomo-Garo et al. have investigated the alterations in the endocannabinoid signaling system in the basal ganglia during the progression of the disease in a transgenic PD mouse model expressing the G2019S mutation of leucine-rich repeat kinase 2 (LRRK2), focusing on the CB2 receptor, and have also analyzed the effects of cannabinoids that selectively target the CB2 receptor in this model [62]. Conversely to the previous studies that have found an up-regulation of CB2 receptors in PD models, they found no alterations neither in the status of the CB2 receptor nor in other elements of the endocannabinoid signaling in the basal ganglia [62]. However, the selective activation of the CB2 receptor with the selective CB2 receptor agonist HU-308 (5 mg/kg, i.p.) improved the most severe behavioral abnormalities present in LRRK2 transgenic mice, which consisted in the reduction in motor strength [62]. Moreover, along with this improvement, a normalization in the impaired autophagy found in these mice was observed [62]. Hence, these data suggest that the CB2 receptor might be a potential pharmacological target in LRRK2 transgenic mice [62]. 

In addition, a study conducted by Kelly et al. has shown that, although the intranigral delivery of the adeno-associated viral vector overexpressing α-synuclein (AAV-α-synuclein) in the rat brain induced extensive overexpression of human α-synuclein in the nigrostriatal pathway, there were no differences in CB1 or CB2 receptor expression in the nigrostriatal pathway [63]. 

Instead, Shi and colleagues have also explored the role of a selective CB2 receptor agonist in a PD animal model [64]. In particular, they have investigated AM1241 (from 0.75 to 12 mg/kg, i.p.), on the neurotoxicity induced by MPTP in a PD mouse model and they have shown that AM1241 significantly inhibited the neural toxicity mediated by MPTP and exerted a significant therapeutic effect on PD mice [64]. Differently from the previous studies, they have found that there was a down-regulation of CB2 receptors in PD mice [64]. After AM1241 administration, the reduced level of CB2 receptor in the brain of PD mice was reversed, the behavior score was considerably elevated and there was also a dose-dependent raise of dopamine and serotonin [64]. Moreover, it has been shown that AM1241 was able to regenerate dopaminergic neurons in PD mice and that the induction of CB2 receptor expression and the raise in phosphorylation of the PI3K/AKT pathway might be the possible mechanisms involved in the neurogenesis effect of AM1241 [64]. 

Moreover, several other studies have also explored the effects of different other selective CB2 receptor agonists in PD animal models.

Chung et al., investigating the mechanisms involved in CB2 receptor-mediated neuroprotection of dopamine neurons in the substantia nigra in the MPTP PD mouse model, have found that treatment with the selective CB2 receptor agonist JWH-133 (10 μg kg^−1^, i.p.) was able to prevent the degeneration of dopamine neurons in the substantia nigra and of their fibers in the striatum induced by MPTP [65]. Interestingly, the neuroprotective action of JWH-133 was found to be associated with the suppression of BBB disruption, astroglial myeloperoxidase expression, infiltration of peripheral immune cells and production of iNOS, as well as various proinflammatory cytokines and chemokines by the activated microglia [65]. Moreover, the non-selective cannabinoid receptor agonist WIN55,212-2 (10 μg kg^−1^, i.p.) mimicked the effects of JWH-133, whereas the CB2 receptor inverse agonist AM630 (20 μg kg^−1^, i.p.) inhibited the neuroprotective effects of JWH-133 and of WIN55,212-2, thus indicating the involvement of the CB2 receptor [65]. Thus, since the activation of the CB2 receptor can inhibit neuroinflammatory processes, BBB disruption, and T-cell infiltration, and can prevent nigrostriatal dopamine neuronal death in the MPTP PD mouse model, targeting the CB2 receptor could be beneficial for PD [65].

Additionally, García-Arencibia et al. have studied the effects of a selective CB2 receptor agonist in a PD animal model [66]. In particular, they have demonstrated that the administration of the selective CB2 receptor agonist HU-308, at a dose of 5 mg/kg, led to a small recovery in a PD rat model, thus suggesting that CB2 receptors could provide some degree of neuroprotection [66]. 

According to this line of research, Javed et al. have explored the CB2 receptor-mediated neuroprotective effects of another selective CB2 receptor agonist, BCP (50 mg/kg, i.p.), in a rotenone-induced PD rat model [67]. BCP treatment reduced the induction of proinflammatory cytokines and inflammatory mediators in rotenone-injected rats [67]. In addition, BCP treatment was able to prevent the depletion of glutathione, inhibit lipid peroxidation, and increase the activity of the antioxidant enzymes superoxide dismutase and catalase [67]. Moreover, it has been shown that BCP prevented the loss of dopaminergic neurons induced by rotenone in the substantia nigra pars compacta and striatal dopaminergic fibers [67]. Of note, the prior administration of the CB2 receptor inverse agonist AM630 (1 mg/kg, i.p.), reduced the beneficial effects of BCP [67,68]. Since it has been shown that the anti-inflammatory and antioxidant effects of BCP in the rotenone-induced PD rat model were mediated by the activation of CB2 receptors, it should be considered that CB2 receptors could be a promising therapeutic target for PD and that BCP might represent a potential interesting molecule for PD treatment [67].

Yu et al. have also evaluated the effects of a selective CB2 receptor agonist in a PD animal model [69]. In particular, they have investigated the effects of GW842166x against dopamine neuron loss and the associated motor deficits in a PD mouse model obtained with 6-OHDA [69]. They have shown that GW842166x (1 mg/kg, i.p.) exerted neuroprotective effects against 6-OHDA-induced neurodegeneration and motor function deficits and that these neuroprotective effects were prevented by the co-treatment with the CB2 receptor inverse agonist AM630 (10 mg/kg, i.p.), thus indicating a CB2 receptor-dependent mechanism [68,69]. In particular, they found that the GW842166x reduced the action potential firing of substantia nigra pars compacta dopamine neurons by decreasing the activation of hyperpolarization-activated cyclic nucleotide-gated (HCN) channel-mediated currents [69]. Hence, GW842166x could exert protective effects against dopamine neuron degeneration by decreasing the action potential firing and the associated calcium influx/load [69]. Considering that it has been shown that GW842166x was safe and well-tolerated in a clinical trial, the results from this study suggest that the use of GW842166x or of other CB2 agonists could be a potential therapeutic strategy to slow disease progression in the early phase of PD [69,70]. 

Moreover, the study conducted by He et al. has also investigated the effects of another selective CB2 receptor agonist in a PD animal model [49]. In particular, in addition to the above described in vitro experiments, He et al. have also analyzed the effects of the selective CB2 receptor agonist AM1241 in a mouse model of PD obtained with MPTP, using CB1 receptor knockout (CB1-KO), CB2 receptor knockout (CB2-KO) and WT mice [49]. They have shown that treatment with AM1241 (6 mg/kg) induced motor function recovery, augmented the release of neurotransmitters, ameliorated the survival of tyrosine hydroxylase + dopaminergic neuron, and induced also high mRNA expression of Pitx3, Nr4a2, and Park2, in CB1-KO animals and WT animals treated with MPTP [49]. On the other hand, these effects did not occur in CB2-KO mice [49]. Hence, the neuroprotective effects of AM1241 could be mediated by CB2 receptors [49]. 

A different area in this field has been explored by Binda et al., who have investigated the effects of exercise on the nociceptive threshold in a unilateral rat PD model induced by 6-OHDA and have also explored the expression of cannabinoid receptors in areas of the central nervous system implicated in the nociceptive process, including the periaqueductal gray matter, anterior cingulate cortex, and thalamus [71]. They found that the treadmill exercise promoted antinociceptive effects and could be involved in the modulation of the nociceptive threshold by increasing the expression of CB1 and CB2 receptors in the periaqueductal gray matter and of CB2 receptors in the anterior cingulate cortex [71]. 

Another interesting approach was that of Ternianov et al., who have analyzed the involvement of CB2 receptors in the neurochemical and behavioral changes caused by the injection of 6-OHDA in mice overexpressing the CB2 receptor (CB2xP) and WT mice [72]. Noteworthy, they have found that the overexpression of CB2 receptors attenuated motor impairment and dopaminergic neuronal loss and also decreased the recruitment of microglia and astrocytes to the lesion, as well as reduced the level of different oxidative parameters [72]. Hence, CB2 receptors can provide neuroprotection against dopaminergic injury [72].

Considering all the studies in PD animal models above described, it should be noted that most of the studies have shown an upregulation of CB2 receptors in different PD animal models [56,57,58,59,61]. However, one of the studies has found no alterations in the status of the CB2 receptor in a transgenic PD mouse model and another one has shown that after viral-mediated α-synuclein overexpression in the rat brain there were no differences in CB2 receptor expression [62,63]. In addition, one of the discussed studies has found a down-regulation of CB2 receptors in PD mice [64].

Of note, all the studies have shown that CB2 receptor agonists exert protective effects in PD animal models. Interestingly, these protective effects seem to be mostly associated with the inhibition of microglial activation/infiltration and of different neuroinflammatory processes [58,61,64,65,67].

Moreover, the overexpression of CB2 receptors has been shown to exert neuroprotective effects, whereas as regards the genetic ablation of CB2 receptors, there are conflicting results [72]. Indeed, it has been shown that the genetic ablation of CB2 receptors in PD animal models can increase the sensitivity to MPTP-induced systemic toxicity and to LPS-induced lesions [58,59]. However, other studies have shown that CB2 receptor-deficient mice were equally susceptible to MPTP and to 6-OHDA [59,60].

### 3.3. Studies in Animal Models of Levodopa-Induced Dyskinesia (LID)

Several studies have investigated the CB2 receptors in models of LID.

Rojo-Bustamante et al. have investigated whether there were differences in the expression levels of the endocannabinoid system elements that could be involved in LID, analyzing parkinsonian monkeys treated with levodopa to induce dyskinesias [73]. They have shown that the expression of CB1 receptors and 2-arachidonoyl glycerol synthesizing/degrading enzymes was altered in basal ganglia during the active phase of LID, whereas the expression of CB2 receptors did not change [73]. 

Rentsch et al. have analyzed the effects of the selective CB2 receptor agonist HU-308 on LID in a mouse model of PD and LIDs, obtained with 6-OHDA and the subsequent treatment with levodopa [74]. They found that HU-308 (1 mg/kg, 2.5 mg/kg, or 5 mg/kg) dose-dependently decreased LID, showing an efficacy comparable to the frontline treatment amantadine [74]. In particular, HU-308 at a dose of 2.5 mg/kg and at a dose of 5 mg/kg decreased dyskinesia in a similar manner, superior to that observed with HU-308 at a dose of 1 mg/kg [74]. Thus, the dose of 2.5 mg/kg of HU-308 was an effective dose that obtained the maximum decrease in abnormal involuntary movement in mice [74]. In addition, it has been shown that the CB2 receptor inverse agonist SR144528 (1 mg/kg) blocked the anti-dyskinetic effect of HU-308 (2.5 mg/kg) and that the combined treatment with HU-308 (2.5 mg/kg) and amantadine (40 mg/kg) had a stronger anti-dyskinetic effect compared to the treatment with HU-308 alone [54,74]. Moreover, it has been observed that HU-308 and amantadine, both administered alone or as co-treatment, reduced striatal neuroinflammation [74]. These data overall suggest that the use of CB2 agonists as potential therapies for LIDs in PD patients should deserve to be studied [74]. 

According to this line of research, Espadas et al. have explored the anti-dyskinetic potential of Δ^9^-THCV (2 mg/kg, i.p.) which has been shown to be neuroprotective in experimental PD in part thanks to its CB2 receptor agonist properties, in an animal model of parkinsonism associated with dopaminergic deficiency, the Pitx3^ak^ mutant mice [75]. They have shown the anti-dyskinetic potential of Δ^9^-THCV to improve adverse effects of levodopa, both delaying the occurrence and reducing the intensity of the dyskinetic signs [75]. Interestingly, one of the possible options to clarify the potential mechanisms underlying the anti-dyskinetic effect of Δ^9^-THCV could depend on the activation of CB2 receptors, for which this compound is an agonist [75]. However, this option should be confirmed by further experiments using Δ^9^-THCV along with a selective CB2 receptor antagonist [75]. 

Overall, it has been shown that there were no changes in the expression of CB2 receptor in the basal ganglia during the active phase of LID and that both HU-308 and Δ^9^-THCV can exert anti-dyskinetic effects in animal models [73,74,75].

### 3.4. Studies Investigating CB2 Receptor Heteromers in Animal Models of PD and LID

Although GPCRs were first considered to function as singular units, to date it has been shown that different GPCRs can function in complex with receptors of the same or different types [76]. The oligomerization of GPCRs can influence ligand binding and receptor coupling to signaling pathways (constitutively but also in response to ligand), and the subcellular localization basally and after ligand interaction [76]. In particular, it has been shown that CB2 receptors can form different heteromers, including: CB1-CB2, CB2-CXCR4, CB2-GPR55, CB2-GPR18, CB2-A2A, and CB2-HER2 [76].

Interestingly, the CB2 receptor heteromers in PD and LID have also been explored. In particular, Martínez-Pinilla et al. have evaluated the striatal expression of heteromers consisting of GPR55 and CB1/CB2 receptors in parkinsonian macaques, with and without LID, and controls, with the aim to discover whether Parkinsonism correlates with altered expression of the heteromers CB1-GPR55_Hets and/or CB2-GPR55_Hets [77]. Noteworthily, they have shown that the expression of both heteromers was significantly increased in caudate, putamen, and accumbens nuclei of parkinsonian animals and returned to normal levels after LID [77].

## 4. Clinical Studies

### 4.1. In Silico and Post-Mortem Studies in PD Patients

Several in silico and post-mortem studies have explored CB2 receptors in PD patients, and we have provided a schematic overview of the most important findings from these studies in Table 1.

As regards the in-silico studies, a human genome wide association studies (GWAS) analysis has shown that the *CNR2* gene is associated with PD [78]. 

As regards post-mortem studies, García et al. have analyzed the post-mortem substantia nigra of PD patients and controls and have found that CB2 receptors were localized in nigrostriatal neurons in the human substantia nigra and that, interestingly, their levels were decreased in the substantia nigra of PD patients, in concordance with the losses of these neurons that occurs in PD [28]. Therefore, the level of CB2 receptors might be used as a potential biomarker for PD [28]. 

Moreover, Grunblatt et al. have found that the CB2 receptor gene expression was down-regulated in the cerebellum and hippocampus of PD patients in comparison with healthy controls [79].

In addition, Gómez-Gálvez et al. have investigated the post-mortem tissues from the substantia nigra of PD patients and controls and have found that CB2 receptors are elevated in microglial cells recruited and activated at lesioned sites in the substantia nigra of PD patients in comparison with controls [61]. Therefore, it has been shown an up-regulation of the CB2 receptors in glial elements from post-mortem tissues of patients with PD [61]. 

Furthermore, Navarrete and colleagues, analyzing the post-mortem brain samples from PD patients and controls, have shown that CB2 receptor brain-specific A isoform (CB2Ar) gene expression was significantly upregulated in the substantia nigra but downregulated in the putamen in PD patients [80]. Moreover, they found that CB2 receptors co-localize with astrocytes but not with neurons or microglial cells in the substantia nigra of PD patients [80]. Hence, these data suggest that the CB2 receptor could be involved in the neuropathological processes of PD [80]. 

Overall, although only a few data are available so far, it should be noted that all the present studies have revealed alterations in the expression of CB2 receptors in post-mortem brain tissues of PD patients [28,61,79,80].

### 4.2. Clinical Trials

To date, there are no clinical trials specifically investigating the role of CB2 receptors or the effects of specific CB2 receptor agonists or antagonists in PD patients [81].

However, there are different trials investigating cannabis-based medicines in PD patients [11,82,83]. Although clinical studies investigating cannabis-based medicine or isolated phytocannabinoids in PD show only a few conclusive data, the use of cannabis-based medicine appears promising [11].

The potential use of nabilone in PD patients seems to be particularly relevant. Nabilone is an analogue of THC that acts as a partial agonist on both CB1 and CB2 receptors in humans, thus mimicking THC effects but showing more predictable side effects and less euphoria [84]. A randomized, double-blind, placebo-controlled, crossover trial conducted by Sieradzan et al. has shown that nabilone can decrease γ-aminobutyric acid (GABA) reuptake in the lateral segment of the globus pallidus (GPl) and ameliorate LID in PD without decreasing the antiparkinsonian actions of levodopa [85]. In addition, Peball et al. have investigated the efficacy and safety of nabilone for the treatment of non-motor symptoms in PD patients in a phase II placebo-controlled, double-blind, parallel-group, enriched enrollment randomized withdrawal trial (NCT03769896) [84,86]. Interestingly, they have shown that the treatment with nabilone was well tolerated and led to an improvement of the overall non-motor symptoms burden, in particular ameliorating anxiety and sleeping problems [86].

## 5. Discussion

To date, PD still represents a clear unmet medical need. Hence, discovering novel preventive and therapeutic approaches and novel potential biomarkers should be a priority of utmost importance in order to improve patients’ quality of life. 

**Figure 2:**
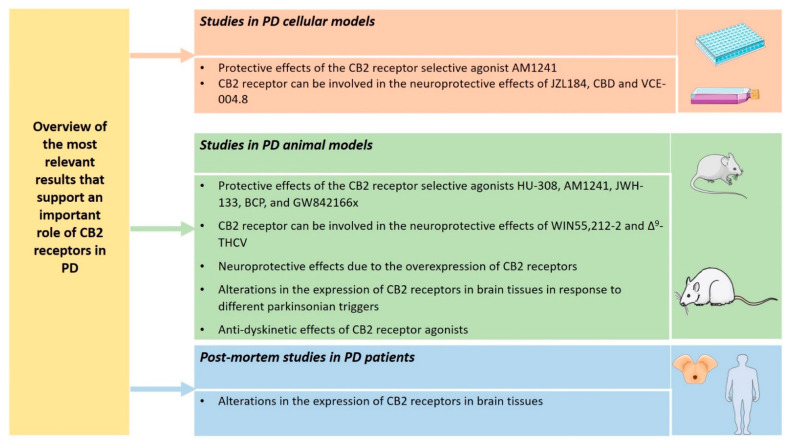
Overview of the most relevant results that support an important role of CB2 receptors in PD. The figure was partly generated using Servier Medical Art (http://smart.servier.com/, accessed on 1 October 2022), provided by Servier, licensed under a Creative Commons Attribution 3.0 Unported License (https://creativecommons.org/licenses/by/3.0/ (accessed on 21 October 2022)).

This review summarizes the current preclinical and clinical studies investigating the CB2 receptors in PD and sheds light on the potential value of CB2 receptors as possible novel biomarkers and therapeutic targets for PD patients. Taking into consideration the studies currently present on this topic, it is worth noting that, although not all the results are convergent, numerous results support the important role of CB2 receptors in PD (Figure 2).

Indeed, it should be considered that results from the preclinical studies in different PD cellular and animal models have shown the protective effects of different CB2 receptor selective agonists, such as AM1241, HU-308, JWH-133, BCP, and GW842166x [49,61,62,64,65,66,67,69]. 

Moreover, other preclinical studies in PD cellular and animal models have shown that CB2 receptor can be involved in the neuroprotective effects of different compounds, such as JZL184, CBD, VCE-004.8, WIN55,212-2, and Δ^9^-THCV [50,51,53,58,59]. 

Furthermore, preclinical studies in PD animal models have shown that, in addition to the pharmacological activation of CB2 receptors, the overexpression of CB2 receptors could also exert neuroprotective effects [72].

It is also worth noting that different preclinical studies in PD animal models have demonstrated alterations in the expression of CB2 receptors in brain tissues in response to different parkinsonian triggers [56,57,58,61].

Moreover, according to certain studies, but not all, the genetic ablation of CB2 receptors in PD animal models can increase the sensitivity to MPTP-induced systemic toxicity and to LPS-induced lesions [58,59]. 

In addition, HU-308 and Δ^9^-THCV have been shown to exert anti-dyskinetic effects in preclinical studies in PD animal models [74,75].

As regards the clinical studies, only a few data are currently available. Although there are different trials analyzing cannabis-based medicines in PD patients, no clinical trials specifically investigating the role of CB2 receptors or the effects of specific CB2 receptor agonists in PD patients are currently present [11,82,83]. 

However, a human GWAS analysis has shown that the *CNR2* gene is associated with PD [78]. Moreover, alterations in the expression of CB2 receptors have been revealed in the post-mortem brain tissues of PD patients [28,61,79,80].

Considering all the discussed studies, it should be noted that the alterations in the expression of CB2 receptors revealed in the brain tissues of PD animal models and PD patients suggest that CB2 receptors might be potential novel biomarkers for PD. In addition, the protective effects of different CB2 receptor-selective agonists in different models of PD suggest that CB2 receptors could be potential novel therapeutic targets for PD.

The absence of central side effects of CB2 receptor-selective drugs, the reduced expression levels of CB2 receptors in the central nervous system, and its inducibility make the CB2 receptor a more interesting target in comparison to the CB1 receptor [30,34]. Hence, the use of selective CB2 receptors agonists might deserve to be further investigated as a potential complementary therapy in PD. Nonetheless, it should be noted that, in general, despite the positive results in preclinical studies of different highly affine ligands targeting CB2 receptors, only a few of them were tested in clinical trials [87]. However, the reasons underlying the difficulties in the clinical development of CB2 receptor ligands are not fully clarified. One of the major obstacles is that, since CB2 receptors are largely expressed in peripheral organs, in particular in the immune system, CB2 receptor ligands could have some side effects, including immunosuppression [34,48]. In addition, a significant obstacle to the translation from animal models to humans could be that the regulation of CB2 receptors could be different across different species [88,89]. Furthermore, inadequate attention to the nuances of CB2 receptor pharmacology could be another potential reason [89]. 

Therefore, the safety and efficacy of the therapeutic targeting of CB2 receptors for PD and LID, the potential differences in the regulation of CB2 receptors between different species, and the mechanisms of action of drugs targeting CB2 receptors should be further investigated, possibly using also other different novel animal models of PD.

Interestingly, it should be also considered that the development of agonists that are signaling pathway-selective and that do not elicit on-target adverse effects has attracted growing interest in recent times [90]. This phenomenon, in which specific pathways are preferred after receptor activation by certain ligands, is indicated by the term “biased signaling” [90]. Recently, it has been suggested that also CB2 receptor ligands could show biased signaling [91]. Despite for the cannabinoid receptors this field is still in its infancy, biased-signaling mechanisms at CB2 receptors could open new paths for the development of improved CB2 receptor-targeted treatment [45,92].

In addition, the value of CB2 receptors as possible biomarkers should be carefully evaluated in PD patients. Genetic studies aiming to investigate the incidence of different polymorphisms of the CB2 receptor gene in PD patients and studies aiming to evaluate the activity of the CB2 receptors in the brain of PD patients with positron emission tomography (PET) imaging techniques are warranted. 

## 6. Conclusions

Overall, further studies are much needed in order to fully clarify the exact role of CB2 receptors in PD and confirm the data on the potential CB2 receptor-mediated neuroprotective action in PD that emerge from the analysis of the studies currently present, thus paving the way to novel possible diagnostic and therapeutic opportunities for PD.

## Figures and Tables

**Table 1:** Schematic overview of the most important findings from the in silico and post-mortem studies investigating the CB2 receptors in PD patients.

In Silico and Post-Mortem Studies in PD Patients	
Results	References
- *CNR2* gene is associated with PD	[78]
- CB2 receptors were found in nigrostriatal neurons in the human substantia nigra - decreased levels of CB2 receptors in the substantia nigra of PD patients	[28]
- CB2 receptor gene expression was down-regulated in the cerebellum and hippocampus of PD patients	[79]
- up-regulation of the CB2 receptors in glial elements from post-mortem tissues of patients with PD	[61]
- CB2 receptor A isoform (CB2Ar) gene expression was significantly upregulated in the substantia nigra but downregulated in the putamen in PD patients - CB2 receptor co-localize with astrocytes but not with neurons or microglial cells in the substantia nigra of PD patients	[80]

## Data Availability

Not applicable.
